# Estimation of Genetic Parameters for Peak Yield, Yield and Persistency Traits in Murciano-Granadina Goats Using Multi-Traits Models

**DOI:** 10.3390/ani9070411

**Published:** 2019-07-01

**Authors:** Judith C. Miranda, José M. León, Camillo Pieramati, Mayra M. Gómez, Jesús Valdés, Cecilio Barba

**Affiliations:** 1 Department of Animal Production, Faculty of Veterinary Sciences, University of Cordoba, 14071 Córdoba, Spain; 2 Department of Genetics, Faculty of Veterinary Sciences, University of Cordoba, 14071 Cordoba, Spain; 3 Department of Veterinary Medicine, University of Perugia, via S. Costanzo 4, 06123 Perugia, Italy; 4 Department of Animal Science, University of Concepcion, Concepcion 4070386, Chile

**Keywords:** accuracy, breeding-values, correlations, heritability, lactation

## Abstract

**Simple Summary:**

The Murciano-Granadina goat is a local breed of importance, not only for the economic and social impact of the breeders but also for its conservation. Estimates of the genetic parameters of peak and persistency traits (not commonly used in breeding schemes) using multivariate models is a feasible tool in early lactation (could genetically modify lactation curves) for improving sustainable production in dairy goats, specifically when more data are available. The genetic variability for the parameters of the lactation curve (peak yield, yield and persistency studied traits) is low. The heritability was low to intermediate in all the traits, being between 0.08 for persistency and 0.17 for yield. The genetic correlations were high for peak yield and yield (0.94), indicating that the selection for both peak production and persistence is feasible, with no detrimental response in either. Murciano-Granadina should be a guide dairy goat, and the result could provide a general strategy applicable to other local breeds.

**Abstract:**

This paper studies parameters of a lactation curve such as peak yield (PY) and persistency (P), which do not conform to the usual selection criteria in the Murciano-Granadina (MG) breed, but are considered to be an alternative to benefit animal welfare without reducing production. Using 315,663 production records (of 122,883 animals) over a period of 24 years (1990–2014), genetic parameters were estimated with uni-, bi- and multivariate analysis using multiple trait derivative free restricted maximum likelihood (MTDFREML). The heritability (h^2^)/repeatability (r_e_) of PY, yield (Y) and P was estimated as 0.13/0.19, 0.16/0.25 and 0.08/0.09 with the uni-trait and h^2^ of bi- and multi-traits analysis ranging from 0.16 to 0.17 of Y, while that of PY and Y remained constant. Genetic correlations were high between PY–Y (0.94 ± 0.011) but low between PY–P (–0.16 ± 0.054 to –0.17 ± 0.054) and between Y–P (–0.06 ± 0.058 to –0.05 ± 0.058). Estimates of h^2^/r_e_ were low to intermediate. The selection for Y–PY or both can be implemented given the genetic correlation between these traits. PY–P and Y–P showed low to negligible correlation values indicating that if these traits are implemented in the early stages of evaluation, they would not be to the detriment of PY–Y. The combination of estimated breeding values (EBVs) for all traits would be a good criterion for selection.

## 1. Introduction

Over a period of 30 years the Murciano-Granadina (MG) breed has been consolidated in European and international agriculture as one of the main dairy goat breeds based on its population, geographical distribution, quality and production, where practically all milk obtained is destined for the cheese industry. Currently, this Spanish breed of goat is considered the most exported due to its hardiness (adequate adaptation to a new environment) and its extensive grazing capacity; MG is a permanent polyestric animal with high prolificacy and easy milking [1].

The breed has an improvement program that provides genetic parameters to measure milk, fat, protein and dry extract performance criteria that guide selection decisions [1]. Based on the selection criteria, in the last year, there is a growing interest in adding traits, combining information of several economically important traits into an overall selection index, to focus on the lifetime profitability of dairy breeds [2]. 

The estimation of parameters for economically important traits makes it possible to predict direct and correlated selection responses (important traits usually show genetic correlations), improving the selection indexes [3]; being precise and impartial; also avoiding reductions in negatively correlated traits when using a single trait. 

In this instance, using multivariate analyses has a great importance in providing reliable and unbiased estimates of genetic parameters [4] in animal populations; but at the same time having uni-trait estimations allows to control the trustworthiness of multi-traits results and helps to identify any problems with the latter [5].

Previous studies of lactations curves in goats [6,7] have implemented the Spline model, providing a good fit of data taking into account the factors of variation. This model also decreases biased and imprecise estimates; summarizing in a few descriptive parameters the productions before and after the peak yield (PY) and persistency (P) of that lactation. This last mentioned trait has been described as difficult to factor in due to its genetic variability [8].

To our knowledge, the modeling of individual lactation curves of P by Spline and subsequent multi-traits combined analyses for PY, yield (Y) and P traits have not yet been reported in the literature. PY and P traits would constitute a point of support for decision making within the genetic breeding improvement scheme, given the possibility of including them as additional criteria in the future. 

Given this background, the objective of the current study was to estimate genetic parameters, (co)variance components, their correlations (genetic and phenotypic), prediction of genetic values (estimated breeding values—EBVs) and their accuracy of PY, Y and P traits using a multiple trait model. We tested the hypothesis that PY and P traits can be used as new selection criteria in the breeding program of the MG breed using multi-traits models. This information is also useful in increasing lactation length and will be able to provide decisive results that should be taken into account in the near future for breeding programs 

## 2. Materials and Methods 

### 2.1. Data Description

The genealogical information, the records of the milk control (ICAR-A4 system cited by Delgado et al. [1]) and data analyzed in this study were taken from the historical archives of the official program of dairy control for MG goats, of the period between the years 1990 to 2014. The general data file contained a total of 1,918,780 records (315,663 corresponded to lactations of a total of 122,883 goats, belonging to 245 farms; where reference sires provided by the breeding centers are used to create genetic connections among the farms).

An exploratory data analysis, exhaustive review of all lactations and filters (records with null or repeated values; daily yields that exceed 10 kg of milk or that are below 0.2 kg; lactations with less than six records; and data beyond ±3 SD) was applied through the debugging of the database. The final file consisted of 1,349,347 records, which corresponded to 180,872 lactations belonging to 85,404 goats of 229 herds.

The age of the active animals was between four and eight years, where the animals between four and five years of age represented 47.55%; and the range of six and seven years of age 33.68%; there being a significant presence of females with more than eight years of age (17.77%).

### 2.2. Pedigree Information

Pedigree files included a total of 38,756 individuals (effective individuals 36,662 representing 94.4% of the total), for the coding Pedigree Viewer 6.5 computer program developed by Kinghorn and Kinghorn [9] was used. Descriptive characteristics of the pedigree file to build the kinship matrix can be seen in Appendix A (Table A1).

### 2.3. Statistical and Genetic Analyses

A mixed model was applied to analyze the various factors that affect the traits under study. Next, the individual lactation curves were analyzed using the Spline model, which has proved to be the one that provides the best goodness of fit for the MG breed (R^2^ = 0.98, mean squared error (MSE) = 0.0020 without existence of autocorrelation among residuals) than several other common functions (Wood, Cappio-Borlino, Cobby and Le Du, Wilmink and Legendre) [6] and for a better estimation of genetic parameters [10]. The Spline function with one knot used:

*y_t_* = *β*_0_ + *β*_1_*t* + *β*_2_*t*^2^  for *t* ≤ *X*, (1)

*y_t_* = *β*_0_ + *β*_1_*t* + *β*_2_*t*^2^ + *β*_3_ (*t* − *X*)^2^  for *t* > *X*, (2)

where *y_t_* represents the average daily milk production recorded on day t, while *β*_0_, *β*_1_, *β*_2_ and *β*_3_ are the parameters specifically designed to adapt to the shape of the lactation curves due to its flexibility. Meanwhile, *X* is treated as an additional parameter to estimate and represents the day of lactation, where the knot point is produced, that is, where the two polynomial functions are linked. For the resolution of this model, statistical software “R” version 3.2.3 [11] was implemented. 

This allowed us to obtain and identify the knots of the individual lactation curve of PY, Y and P traits. These traits were defined in a lactation curve (standardized to 210 days) as (i) PY is a period of maximum production until day 70, (ii) Y is the general milk yield of a complete lactation and (iii) the final phase of continuous descent in lactation curves where the level at which the production or length of lactation is maintained called P (occurs after PY and is calculated as the difference between the accumulated production of days 71–210 and the average of the initial third of 11–70 days). In fact, Y is currently the object of selection and PY and P are considered candidate traits to be included in the genetic improvement program.

The components of (co)variance for all the random effects were estimated using the multiple trait derivative free restricted maximum likelihood (MTDFREML) programs [12]. The lactation curve traits (PY, Y and P) were analyzed with single-trait (uni-trait), two-trait (bi-trait) and three-trait (multi-traits) models. Starting values for each trait of interest were initially obtained with a univariate analyses, and for the next step to obtain the starting values for the multivariate analyses, a series of combined bivariate analyses was realized.

The final model for the analysis of lactation curve traits included fixed effects of the classical Herd–Year–Season of kidding (HYS) effect (herds = 137; year = 24 from 1990–2014 and season = four levels: Winter, spring, summer and fall), lactation number (LN; four levels: First, second, third and fourth and greater), type of kidding (TK; three levels: Single, twin and triplet and greater) and age of goat (linear and quadratic covariate). 

Animal (additive genetic value), permanent environmental and residual effects were included as random effects. The required convergence criterion of Var [–2log (L)] (where L represents the likelihood function) was <1 × 10^−9^.

The description of the model in matrix notation was:

y = Xb + Z_1_a + Z_2_p + e, (3)

where y = is the vector of phenotypic information for the PY, Y and P traits analyzed; b = vector of fixed effects (HYS, LN, TK and age (covariate included)); a = vector of random additive effects of the animal; p = the vector of the permanent environmental effect; e = is the vector of residuals effects for trait analyzed; X, Z_1_ and Z_2_ = are incidence matrices that are relative to fixed effects, additive genetic effects and permanent environmental effects, respectively.

The calculations of the genetic parameters, the best linear unbiased prediction (BLUP) solutions for the fixed effects (i.e., breeding values) and their accuracies of the animals for PY, Y and P traits, were obtained by solving the mixed model equations same as that used in León [13]. The three traits of interest were analyzed with the effects previously described in model, structured as follows: 

Model uni-trait was used for the univariate analysis: Each trait (PY, Y and P) was studied as a separate trait. The variance-covariance matrix of random effects was structured according to León [13].

Model bi-traits was used for the bivariate analysis: Each pair of traits: (PY–Y, PY–P and Y–P) was independently analyzed. 

Model multi-traits was used for the multivariate analysis: In this instance, three traits (PY, Y and P) were analyzed simultaneously.

Matrix notation models of statistical and genetic analyses:

(4) $$\left[\begin{array}{c} y_{1}\\ y_{2} \end{array}\right]=\left[\begin{array}{cc} X_{1} & 0\\ 0 & X_{2} \end{array}\right]\left[\begin{array}{c} b_{1}\\ b_{2} \end{array}\right]+\left[\begin{array}{cc} Z_{a1} & 0\\ 0 & Z_{a2} \end{array}\right]\left[\begin{array}{c} a_{1}\\ a_{2} \end{array}\right]+\left[\begin{array}{cc} Z_{pe1} & 0\\ 0 & Z_{pe2} \end{array}\right]\left[\begin{array}{c} pe_{1}\\ pe_{2} \end{array}\right]+\left[\begin{array}{c} e_{1}\\ e_{2} \end{array}\right],$$

(5) $$\left[\begin{array}{c} y_{1}\\ y_{2}\\ y_{3} \end{array}\right]=\left[\begin{array}{ccc} X_{1} & 0 & 0\\ 0 & X_{2} & 0\\ 0 & 0 & X_{3} \end{array}\right]\left[\begin{array}{c} b_{1}\\ b_{2}\\ b_{3} \end{array}\right]+\left[\begin{array}{ccc} Z_{a1} & 0 & 0\\ 0 & Z_{a2} & 0\\ 0 & 0 & Z_{a3} \end{array}\right]\left[\begin{array}{c} a_{1}\\ a_{2}\\ a_{3} \end{array}\right]\left[\begin{array}{ccc} Z_{pe1} & 0 & 0\\ 0 & Z_{pe2} & 0\\ 0 & 0 & Z_{pe2} \end{array}\right]\left[\begin{array}{c} pe_{1}\\ pe_{2}\\ pe_{3} \end{array}\right]+\left[\begin{array}{c} e_{1}\\ e_{2}\\ e_{3} \end{array}\right],$$

where *y_i_* = vector of observations for the *i*-th trait; *b_i_* = vector of fixed effects that contains the effect of HYS (herd–year–season), LN and TK to the *i*-th trait; *a_i_* = vector of direct additive genetic effects for the *i*-th trait; p_i_ = vector of permanent environmental effects for the *i*-th trait; *e_i_* = vector of residual effects for the *i*-th trait and *X_i_* and *Z* are design matrices that relate the data using fixed and random effects, respectively.

The (co)variance structure of the random effects (permanent environmental, animal and error effects) for the models were structured and designed according to León [13] and Espinoza-Villavicencio et al. [14], respectively.

The parameters of *h*^2^ and *r_e_* were calculated by the following formulas:

(6) $$h^{2}=\frac{\sigma^{2}a}{\sigma^{2}p}\;r_{e}=\frac{\sigma^{2}a+\sigma^{2}pe}{\sigma^{2}p},$$

where *h*^2^ is the heritability; *r_e_* is the repeatability; $\sigma^{2}a$ is the direct additive genetic variance, $\sigma^{2}pe$ is the permanent environmental variance related to repeated records and $\sigma^{2}p$ is the total phenotypic variance that includes the sum of the $\sigma^{2}a$ plus $\sigma^{2}pe$ and residual variance ($\sigma^{2}e$).

Standard errors were estimated using the procedure cited in León [13], which is included in the MTDFREML program. 

The estimates of genetic (*r_g_*), and phenotypic (*r_p_*) correlations on all pairwise combinations of traits (bi-traits analysis) were obtained from the estimation of covariance components using the following formulas:

(7) $$r_{g}=\frac{\sigma_{aij}}{\sqrt{{\sigma^{2}}_{ai}\;{\sigma^{2}}_{aj}}}\;r_{p}=\frac{\sigma_{pij}}{\sqrt{{\sigma^{2}}_{pi}\;{\sigma^{2}}_{pj}}},$$

where *σ_aij_* = additive genetic covariance between trait *i* and *j,* *σ^2^_ai_* = additive genetic variance for trait *i,* *σ^2^_aj_* = additive genetic variance for trait *j*; *σ_pij_* = phenotypic covariance between traits *i* and *j*, *σ^2^_pi_* = phenotypic variance for trait *i* and *σ^2^_pj_* = phenotypic variance for trait *j*. 

To evaluate how likely it is that the EBVs are correctly predicted; the accuracy level and average reliability of the EBVs for the traits were calculated. On the other hand, in order to test results of quality control, we considered the criterion of invariability of estimate from uni-trait to multi-traits models.

## 3. Results

### 3.1. Descriptive Statistics

Basic descriptive statistics obtained for the traits PY, Y and P are shown in the Table 1. The coefficient of variation exceeded 24 percent for each of the three traits.

In the multi-traits analyses no problems with convergence or with impermissible values were encountered. The estimates of the components of variance and genetic parameters were consistent and are shown in Table 2.

### 3.2. Variance Components

The estimated genetic variance (**σ^2^*_a_***) according to uni-, bi- and multi-traits analyses were higher for PY and Y (ranged from 0.014 to 0.015) than for P (constant value of 0.005 in all models). The variance of permanent environmental (**σ^2^*_pe_***) effects (Table 2) was higher for PY and Y (oscillating between 0.007 to 0.009) than for P, which showed a substantially lower value of 0.001. Results for residual (**σ^2^*_e_***) and phenotypic (**σ^2^*_p_***) variances also showed oscillations, where PY and Y had increased estimates in bi- and multi-traits analyses compared to the uni-trait estimates for P was constant across all analyses.

For all these traits it is assumed that each control is a repeated measure of lactation and each lactation was analyzed in an individualized manner. In general, it is emphasized that the estimated genetic parameters for the PY, Y and P traits using all three analyses were similar due to the little variation of the components of variance. 

### 3.3. Heritability and Repeatability Estimates

In a general way, the estimated h^2^ obtained by MTDFREML for the traits in all three analyses were low and similar in values (Table 2). The average h^2^ showed slightly higher values for PY (0.13) and Y (0.17) than for P (0.08).

However, in this table, it can be noted that in the multivariate analysis (bi- and multi-traits) for Y trait the h^2^ was slightly increased; whereas the other traits (PY and P) maintained a constant h^2^ with respect to uni-trait analysis. 

The r_e_ for the P and PY traits in the three analyses was low (0.09 to 0.10 and 0.19 to 0.21 for P and PY traits, respectively). However, r_e_ of bi-trait (PY–Y combination) and multi-traits (PY and Y traits) analysis confirmed a slight increase, due to oscillations shown in their values with respect to uni-trait analysis. In turn, the r_e_ of the P trait in uni- and bi-traits was maintained, but in multi-traits showed a slight increase (Table 2).

### 3.4. Genetic and Phenotypic Correlations

The genetic and phenotypic correlations are shown in Table 3. The combinations of traits as PY–Y, PY–P, Y–P and PY–Y–P show oscillations ranging from –0.05 and 0.94 in the case of genetic correlations and between –0.071 and 0.441 for phenotypic correlations for bi-multi-traits. PY–Y traits were highly correlated (positively), while PY–P and Y–P traits had low to negligible (negative) correlation values. As soon as the phenotypic correlations were observed, the PY and Y traits with P showed significant differences.

The use of multi-traits analysis with respect to uni- and bi-trait analysis changed the maximum of EBVs obtained for all traits (Table 4), while in the minimum values only the PY trait presented changes. The accuracy levels of the EBVs for all the traits were superior to 0.93 at its maximum levels (the minimum values in all traits were 0). It can be seen that in the PY–Y analysis they were increased and the reliability level for all the traits was shown to be between 36% to 47%.

## 4. Discussion

In the present study, PY and P traits were used practicably as a new selection criterion into the breeding program of the MG breed using multi-traits models. This research indicates that the animal model of repeatability allowed the estimation of the components of variance and (co)variance, genetic parameters, genetic and phenotypic correlations, accuracy breeding value for the PY, Y and P traits using uni-, bi- and multi-traits analysis with full convergence and consistent results. In fact, invariability of the estimation from uni- to multi-traits models suggests a good signal as quality control of results. 

To the best of our knowledge, this is the first study where the PY and P traits (traits of economic interest) are evaluated together with selection object traits of the program (e.g., Y). The multi-traits analysis is suggested to analyze traits that have not been considered as selection criteria (i.e., PY and P) but that belong to a population that has been subjected to selection by other traits (e.g., Y). According to Baselga [15], there is the question related to the bias that can be incurred when analyzing these traits that have not been through the selection criteria in a uni-trait. Therefore, in order to avoid such an occurrence, it is necessary that the traits of interest should be analyzed together with those that intervened in the selection decisions.

### 4.1. Variance Components

The results of the variance components estimates (VCE) from the uni-trait model until the simultaneous analyses showed good convergence and were similar. This is in agreement with Meyer [5], who indicated that having uni-trait estimates thus provides a check on multi-traits results, and helps identify any problems with the latter. Nevertheless, the more traits that are analyzed simultaneously, the more computational effort (i.e., the need for random access memory can grow with the square of the number of traits and the computation with the cube).

The variance component denotes that there are traits predominantly influenced by environmental effects, where the σ^2^*_a_* contributes little to the total variance. Consistent with this, it should be taken into account that the environment does not directly modify the genetic constitution of the individual, but it does determine the extent to which it is expressed, the genetic potential of the animals expressed and their respective interactions.

The σ^2^*_a_* obtained in uni-, bi- and multi-traits analyses, was low, indicating a greater impact of the other variances (e.g., σ^2^*_pe_*). This evidence was corroborated by Oldenbroek and van der Waaij [16] and Espinoza-Villavicenio [14], who discussed that small σ^2^*_a_* effects indicate that the traits are predominantly influenced by environmental factors, such as nutrition, climate, exposure to diseases, factors that vary from one farm to another or between individual animals on the same farm, and by the contributions of the dominant or epistatic genes.

### 4.2. Heritability and Repeatability Estimates

The low h^2^ estimates for traits using uni-, bi- and multi-traits analyses (ranging from 0.08 to 0.17), suggest that these are complex traits, which remain stable for several generations. This means a slow and often unstable selective effort and reaffirms the great environmental influence and not only the possible genetic variations existing between animals.

Although these traits have low h^2^ values, these can be measured if there is available information on a large number of animals; as this is economically important for farmers, the evaluation of these traits was justified. Oldenbroek and van der Waaij [16] pointed out that, if a huge number of offspring can be produced, such as in dairy animals, the EBV can be estimated more accurately. In this regard, Ayalew et al. [4] mentioned that the traits will be affected by many pairs of genes and by the environment; to which the latter contributes to the high σ^2^*_e_*.

The estimates of h^2^ (0.13) obtained for the PY trait in MG goats were low and consistent between the three models. In turn, to the best of our knowledge there are no reports of h^2^ in the literature for this trait in MG goats. In this regard, a report of Pollot and Gootwine [17] on this trait in small ruminants describes a h^2^ of 0.12, placing our results close to that report in sheep. Nevertheless, Devendra and Liang [18] suggest that the biological efficiency of the goat distinguishes it from other ruminants because of its high digestive efficiency due to the good conversion of food from non-competitive sources, resulting in efficient milk production.

In relation to this, Oliveira et al. [19] indicate that this trait is very susceptible to environmental factors, which can lead to greater variations of error in different phases of lactation; and even between groups of some animals of intensive production (animals with higher production and shorter lactations) and of extensive production (animals with high P of lactation).

Regarding the h^2^ of the Y trait, Kominakis et al. [20] stresses that this trait has been studied extensively, presenting various estimates in different goat breeds over time (range of estimates in the first descriptions ranged from 0.17 to 0.68).

In the last decade, the reports continue to show variations such as the studies of Thepparat et al. [21] with several breeds of goats and their crosses (Saanen, Anglo-Nubia, Toggenburg, Alpina and goats native to Thailand) establish h^2^ ranges from 0.11 to 0.30 with four Spline knots. García-Peniche et al. [22] with several goat breeds (Alpina, La Mancha, Nubian, Oberhasli, Saanen, Toggenburg) also concluded that the estimated h^2^ for all of them is 0.35.

In our study, we found that the estimates of h^2^ for the Y trait in MG goats were 0.16 (uni- and bi-traits analysis) to 0.17 (multi-traits analysis). These results are within the ranges described in previous studies conducted on the breed that report a lower to intermediate h^2^ with complete and standardized milk yields at 210 and 240 days, respectively. León and Delgado et al. [1,13] described a 0.14 to 0.21 of h^2^; while an even lower h^2^ was reported by Gonzalez-Peña [23] in the breed from 0.06 to 0.11 (with different models).

The h^2^ of P trait in MG goats were the lowest (0.08) in the three models analyzed. This value was below the reported Menéndes-Buxadera et al. [24] in the MG breed with an estimated h^2^ of 0.20 performed with a random regression analysis for the first and second lactation in milk yield between week 17 and 35 in lactation.

However, our results are closer to the results reported by González-Peña [23] in MG analyzed by five persistency measures (modifying the formulas respectively); where intervals were described with estimates from 0.06 to 0.13. In the literature, several estimates are also reported, such as those described by De Oliveira-Menezes et al. [25] in goats of the Saanen breed with values of 0.03 to 0.09 and in Payoya goats with values from 0.26 to 0.40 cited by González-Peña [23].

The diversity of estimates for measures of P in goats are also present in studies of reference for dairy cattle, where a large range of h^2^ estimates with different types of models (values between 0.01 to 0.32) respectively was found by [26,27,28].

All this variability of results is due in part to the different methodologies used, but we believe that an independent analysis of each lactation curve in different lactations by goats will bring us closer to the “real” value of P to have a measure of biological efficiency. Thereafter, the genetic relationship with other important traits such as the somatic cell score, reproductive traits and longevity would be analyzed. In this regard, Arnal et al. [8] highlights that the P plays a key role in the discrimination of lactation curve types.

The importance of this trait is defined by having a flatter lactation curve without a decrease in the total production (desirable). Its economic value in itself, due to its favorable correlation with factors as health [27] and fertility of the female [29] would suggest a flexible scope for modifying lactation curves via genetic selection [30]. Hence this would be helping milk producers in the effective management of feeding and reproduction during early lactation.

In the current study, the r_e_ estimates for lactation curve traits using uni-, bi- and multi-traits analysis were low for PY and P traits (between 0.09 and 0.20), whereas the Y trait had a medium r_e_ (about 0.3). For the Y trait using the multi-traits vs. uni-trait analysis, both parameters (h^2^ and r_e_) increased slightly. Estimates for the Y trait were similar to the previous reports for the MG breed [13], but are not in agreement with González-Peña [23] who describe that r_e_ tends to be twice the h^2^. Nevertheless, in the case of PY and P traits, no r_e_ reports were found in the literature for MG goats. 

The parameter of r_e_ is the maximum limit of h^2^, and their calculation is necessary because although it does not indicate genetic variation, it does allow us to establish a limit to it and will also indicate the relationship of how many records of the same trait must be obtained at different times of the animal’s life, before being discarded [16].

### 4.3. Genetic and Phenotypic Correlations

The genetic correlations observed for PY–Y traits were large (positively with values very close to 1.00 i.e., estimate of 0.94), while PY–P and Y–P traits showed low to negligible (negative) correlation values. Consistent with this, Falconer and Mackay [31] suggests that this correlation expresses the degree to which these two measures are genetically reflected; becoming practically the same trait.

Analyzing more dairy traits (such as PY, Y and P) as a whole and their correlations, allows us to adequately weigh the possible negative indirect genetic responses between traits and facilitate its use to rank animals and make breeding decisions [3].

This positive genetic correlation between traits also indicates that an improvement of one trait has a positive impact on the other [4], and that they are functionally related traits. In relation to this, the results suggest that it is better to continue selecting the animals by the Y trait. In turn, the analysis of the P trait needs to be interpreted with caution.

A current study with Alpinas and Saanen goats corroborate that estimates of higher genetic correlations will be seen in estimates at shorter intervals [19]. Our results are consistent with these studies because PY is adjacent to the Y and the latter will be increased or decreased according to the intensity of PY. Besides, the most notable investigation with Alpine goats indicate that the genetic correlations appear smaller as the Y is increased and the explanation would be by groups of genes, since the expression of those genes are related to milk production and its variations throughout lactation [7].

As for the evidence for a strong association between traits, Oldenbroek and van der Waaij [16] points out that strongly associated traits would indicate that the genes that affect them operate in a common physiological way. Even some papers report correlations of 0.98 to 1.00 [32] or of 0.21 to 0.95 [7] for the daily yield in different test days with two evaluation models.

Oldenbroek and van derWaaij [16] describe that since there is a permanent high genetic correlation (because there is no crossing with other breeds), we could be in the presence of a pleiotropic effect. Therefore, this would reaffirm the performance of multi-traits analysis in improvement programs [4]. In addition, if there were unreliable estimates or very small correlations close to zero, they would validate a legitimate justification for not using a multi-traits analysis in the schemes.

The genetic correlations obtained for PY–P and Y–P traits (negative) in goats, to the best of our knowledge were not reported but was described in other ruminants. For instance, a study in buffaloes have provided evidence for the correlations of PY–P, where mean correlations (–0.24) was reported by Pareek and Narang [33]. This reference could suggest that animals with high P can maintain their dairy yields or otherwise indicate that the higher and earlier the PY, the greater is the increase in the Y of lactation and the shortening of its P.

Therefore, given that the correlations obtained in this study are smaller, we advise caution in the selection for these traits, because it may bring antagonistic responses at certain points along the length of lactation. Nevertheless, in sheep, a previous study indicates this type of negligible correlations, suggesting a flexible scope for modifying lactation curves via genetic selection [30].

Regarding the phenotypic correlations, the PY–Y traits showed good (positive) correlation. Consistent with this, Falconer and Mackay [31] describe that when there are low h^2^ as in our results, these phenotypic correlations will be caused mainly by the environmental correlation. In the case of PY–P traits, they showed a phenotypically low correlation (negative), indicating a low antagonism between these traits.

In dairy cattle, the phenotypic correlations between Y and P were not significant [34]. Therefore, globally this explains that these traits are influenced by more or less the same genetic and environmental factors.

Furthermore, the accuracies calculation for PY, Y and P traits shows stable to slight improvement values from the uni-trait model to multi-traits model. Consequently, the multi-traits analyses will improve accuracies by a better connection between data due to the residual covariance between traits [15].

This enhanced accuracy is due to the ability of the model to account for the relationship between the traits and better connections in the data due to residual covariance between traits [4]. Likewise, multivariate models gave better reliabilities than univariate models in the prediction of genetic merit, which is mainly a result of the model’s ability to use extra information from correlated traits.

Finally, this study provides evidence that PY and P (traits of interest), evaluated in conjunction with criteria object of selection (i.e., Y) using a repeatability model, can be used in the future as a point of support in making decisions about the criteria of selection within the scheme of selection of the MG breed. In future investigations, it is also suggested to evaluate the PY and P traits in combination with other measures (e.g., day at peak, % fat, % protein and % dry extract).

Even for lower values of h^2^ such as the P trait, the possibility of optimally selecting the lactation curve [30] is suggested. In fact, if the objective is to genetically modify the characteristics of the lactation curves (PY, day to peak and P), precise parameter estimates with less bias are needed for individual lactation curves.

## 5. Conclusions

The lowest levels of additive genetic variability for PY, Y and P, were estimated in the MG goat breed. The h^2^ estimates were low and close to intermediate for all traits, indicating that the major part of variation for those traits were due to environmental factors. Therefore, this suggests that these traits are very complex and remain quite stable for several generations. The r_e_ estimates for PY and P traits were low confirming that these traits are mainly influenced by managerial and temporary environmental effects, whereas the estimates for the Y trait had a medium value. 

Even, for the Y trait using the multi-traits vs. uni-trait analysis, both parameters (h_2_ and r_e_) increased slightly. Genetic correlations between PY and Y were large and positive, indicating that it is possible to select for both traits, whereas PY–P and Y–P traits showed low to negligible and negative correlations. The phenotypic correlations indicated low antagonism and levels of significance.

These results indicate that there is no antagonism between the traits under study and that they can be combined in the early stages of evaluation of the selection program, with careful interpretation of the P trait analysis. The accuracies calculation for the lactation curve traits (PY, Y and P) shows more stable values from the uni-trait model to multi-traits model. Therefore, this suggests that multi-traits analysis will improve accuracies by a better connection between data due to residual covariance between traits.

## Figures and Tables

**Table 1:** Descriptive statistics for PY (kg/day), Y (kg/day) and P (dimensionless) traits in Murciano-Granadina goats.

Traits	*N*	Mean	SD	CV (%)
PY ^a^	34193	1.21	0.35	29.00
Y ^b^	36497	1.05	0.32	30.33
P ^c^	38206	1.03	0.25	24.47

PY, peak yield; Y, yield; P, persistency; *N*, number of observations and SD, standard deviation. ^a,b,c^ combinations of superscripts are used for the analyses bi-traits.

**Table 2:** Estimates of variance components (additive genetic (*σ^2^a*), permanent environmental (*σ^2^pe*), residual (*σ^2^e*) and phenotypic variances (*σ^2^p*)), heritability (*h*^2^) and repeatability (*r_e_*) for PY, Y and P traits estimated by uni-, bi- and multi-traits models in Murciano-Granadina goats.

Model						
Uni-trait						
Traits	*σ^2^a*	*σ^2^pe*	*σ^2^e*	*σ^2^p*	*h^2^* + SE	*r_e_* + SE
PY	0.014	0.007	0.088	0.109	0.13 ± 0.02	0.19 ± 0.04
Y	0.014	0.008	0.065	0.087	0.16 ± 0.02	0.25 ± 0.04
P	0.005	0.001	0.056	0.062	0.08 ± 0.01	0.09 ± 0.01
Bi-traits						
PY ^a^	0.015	0.008	0.091	0.115	0.13 ± 0.01	0.20 ± 0.02
Y ^b^	0.015	0.008	0.066	0.089	0.17 ± 0.02	0.26 ± 0.03
PY ^a^	0.014	0.007	0.088	0.109	0.13 ± 0.01	0.19 ± 0.02
P ^c^	0.005	0.001	0.056	0.062	0.08 ± 0.01	0.09 ± 0.01
Y ^b^	0.014	0.008	0.065	0.087	0.16 ± 0.02	0.25 ± 0.03
P ^c^	0.005	0.001	0.056	0.062	0.08 ± 0.01	0.09 ± 0.01
Multi-traits						
PY	0.015	0.009	0.092	0.116	0.13 ± 0.01	0.21 ± 0.02
Y	0.015	0.008	0.066	0.090	0.17 ± 0.02	0.26 ± 0.02
P	0.005	0.001	0.056	0.062	0.08 ± 0.01	0.10 ± 0.01

PY, peak yield; Y, yield and P, persistency. ^a,b,c^ different superscripts indicate the combinations for bi-traits analyses.

**Table 3:** Genetic (above diagonal) and phenotypic correlations (below diagonal) for PY, Y and P traits in Murciano-Granadina goats.

Traits	PY	Y	P
PY		0.94 ± 0.011_b_	−0.16 ± 0.054 _b_
		0.94 ± 0.011_m_	−0.17 ± 0.054 _m_
Y	0.441 ± 0.052		−0.06 ± 0.058 _b_
			−0.05 ± 0.058 _m_
P	−0.071 ± 0.19	0.007 ± 0.17	

PY, peak yield; Y, yield; P, persistency; b, bi-traits and m, multi-traits

**Table 4:** Range of estimated breeding values (EBVs), accuracy and average reliability of EBVs obtained for PY, Y and P traits estimated by uni-bi-multi-traits models in Murciano-Granadina goats.

Model				
Uni-trait				
Traits	Maximum	Minimum	Accuracy	Average reliability (r_ap_)
PY	0.31	−0.32	0.95	0.41
Y	0.31	−0.33	0.96	0.46
P	0.17	−0.13	0.93	0.36
Bi-traits				
PY ^a^	0.33	−0.33	0.96	0.46
Y ^b^	0.33	−0.33	0.96	0.47
PY ^a^	0.31	−0.32	0.95	0.41
P ^c^	0.17	−0.13	0.93	0.36
Y ^b^	0.31	−0.33	0.96	0.46
P ^c^	0.16	−0.13	0.93	0.36
Multi-traits				
PY	0.33	−0.33	0.96	0.46
Y	0.33	−0.33	0.96	0.47
P	0.17	−0.13	0.93	0.36

PY, peak yield; Y, yield and P, persistency. ^a,b,c^ different superscripts indicate the combinations for bi-traits analyses.

## Appendix A

**Table A1:** Descriptive characteristics of the pedigree file.

Pedigree file statistics	*N*
No. of individuals with records	38,756
Effective individuals in the pedigree	36,662
Individuals with only a sire	285
Individuals with only a dam	1359
Individuals with sire and dam	17,904
Number of sires	1659
Number of dams	10,000

*N*, number of observations.
